# A Lifelong Commitment: The Story of Dr. Kisu Song and His Impact on Korean Healthcare

**DOI:** 10.7759/cureus.70022

**Published:** 2024-09-23

**Authors:** Joseph Song, Amelie Oshikoya

**Affiliations:** 1 Osteopathic Medicine, Orlando College of Osteopathic Medicine, Winter Garden, USA; 2 Primary Care, Orlando College of Osteopathic Medicine, Winter Garden, USA

**Keywords:** community service, historical change, historical vignette, history, korean medicine, korean war, medical legacy, rural healthcare

## Abstract

Dr. Kisu Song, an allopathic medical doctor specializing in general surgery, significantly impacted South Korean medicine and healthcare through his lifelong dedication to serving rural communities. Born on December 25, 1939, during the Japanese colonial period, Dr. Song witnessed the tumultuous transformation of Korea from a colonized nation to a divided one. His early life was marked by the Japanese colonial period and the Korean War and its aftermath, which influenced his decision to pursue medicine over law, a choice guided by the pressing needs of his community and his father's legacy. This biography explores Dr. Song's journey from a young student in a war-torn country to a pioneering rural physician in South Korea. His contributions, particularly in underserved areas, exemplify his profound commitment to healthcare and community service.

## Introduction and background

Introduction

Dr. Kisu Song's career is a testament to the profound impact a dedicated physician can have on both individual lives and broader community health. Born during a period of intense historical change in Korea, Dr. Song's life was shaped by the political and social upheavals of his time. His journey from a young boy in a divided Korea to a pioneering general surgeon in rural South Korea illustrates a narrative of resilience, dedication, and selfless service. This biography provides an overview of Dr. Song's life, examining the historical and personal factors that influenced his career, his contributions to medicine, and his lasting legacy.

Background

**Figure 1:**
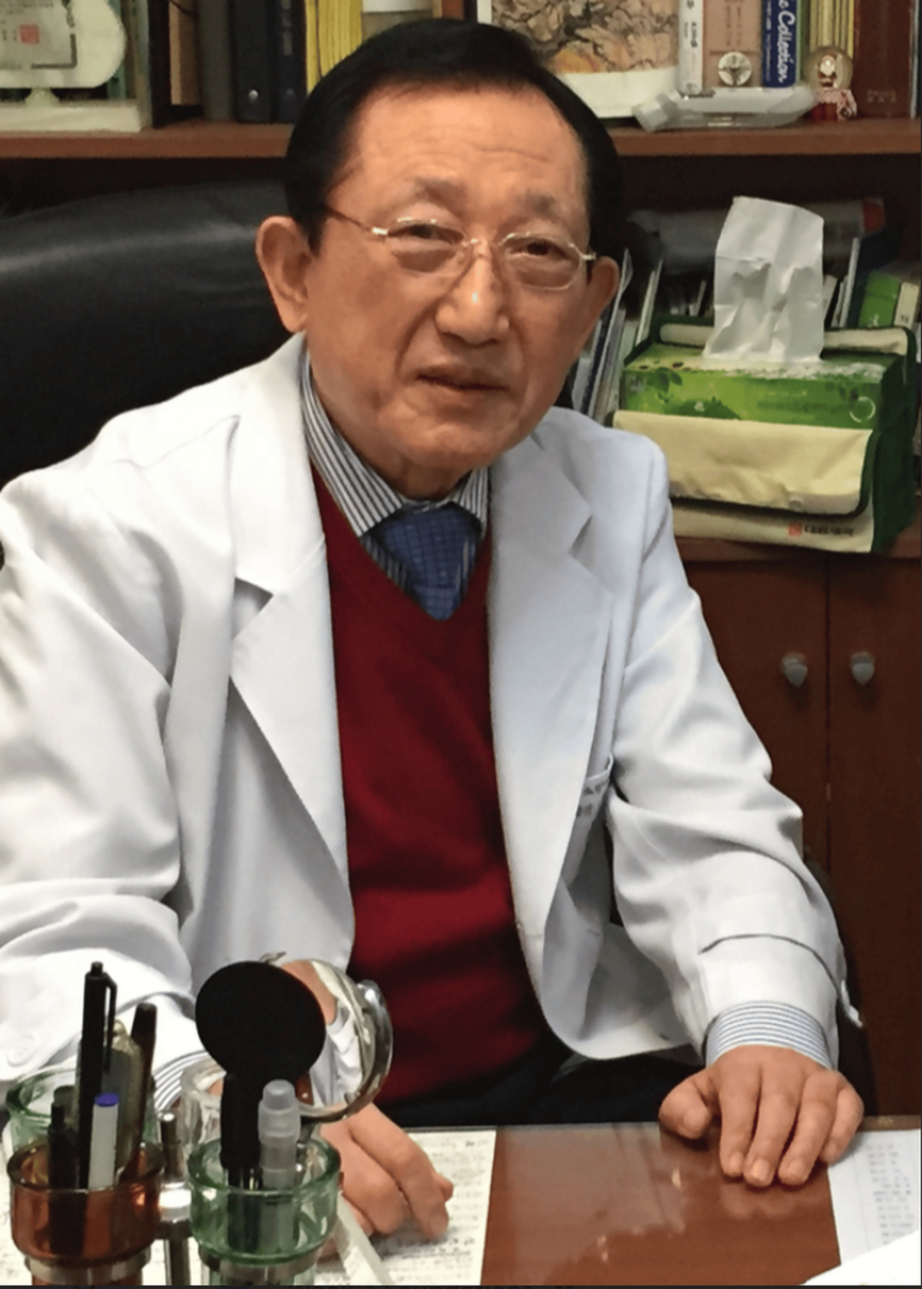
Dr. Song in 2015 at his private clinic.

Dr. Kisu Song (Figure 1) was born on December 25, 1939, during the Japanese colonial period in Korea. The end of World War II in 1945 marked the cessation of Japanese colonial rule and the beginning of Korea's division into North and South [1]. The Korean Peninsula was split along the 38th parallel, with the Soviet Union establishing a communist regime in the North and the United States supporting a democratic government in the South [2]. This division set the stage for the Korean War (1950-1953), which had devastating effects on the Korean people, resulting in approximately 2.5 million casualties and a lasting division between the North and South [2].

Dr. Song's early years were heavily influenced by the socio-political turmoil of this era. His father, a prosecutor, decided to defect from North Korea to South Korea to escape the violent communist regime. The perilous journey across the border, involving evasion of gunfire from North Korean soldiers, was a testament to the family's determination to seek a better future [3]. Despite the ongoing Korean War and its harsh conditions, including rampant infectious diseases, Dr. Song's father encouraged him to pursue a career in medicine. This guidance was rooted in the belief that medicine, unlike law, had universal applicability and was essential in times of crisis.

Dr. Song's academic journey began at the Catholic University School of Medicine in 1958. However, his path was interrupted by military service, as South Korea conscripted men for the army due to the ongoing war. Following his return from the military, Dr. Song observed the significant medical needs of his country, especially in rural areas where healthcare was scarce. Consequently, he decided to open a private clinic in Buyeo, Chungcheongbuk-do, serving as the one of the few healthcare provider for the town. Although his clinic had 25 beds and 8 nurses, and he was the only physician, it functioned more like a hospital, treating both in-patients and out-patients. People came seeking care in various specialties. In the early days of South Korea’s healthcare system, the distinction between ambulatory clinics and hospitals was not clearly defined.

## Review

Dr. Song's impact on healthcare in South Korea is notable for its breadth and depth. In Buyeo, his clinic was more than just a place for surgical care; it was a critical lifeline for the community. A town with fewer than 100,000 residents, Buyeo had only four private clinics. There was no public hospital, only a small public health center, but its capacity to serve the community was far from adequate. In those days, with no public transportation or cars, there was little interaction between clinics, and for many people, his clinic was the only accessible healthcare option available to them. The lack of medical professionals in rural areas meant that Dr. Song had to assume multiple roles: general surgeon, obstetrician, emergency medical doctor, internist, and pharmacist. His practice was characterized by an unwavering commitment to his patients, providing care irrespective of their ability to pay and offering services around the clock.

The significance of Dr. Song's work is underscored by the broader context of South Korean healthcare during his career. After the Korean War, the Korean National Institute of Health began monitoring communicable diseases that had surged during the conflict [3]. Dr. Song's role in combating these diseases and his pioneering efforts in rural healthcare were critical in improving public health outcomes. His clinic in Buyeo played a pivotal role in addressing health disparities between urban and rural areas.

According to the International Medical Community, South Korea's first health insurance law, the Medical Insurance Act, was enacted in December 1963 [4]. Dr. Song began practicing in 1966, making him one of the first-generation physicians in the nation, particularly in rural areas. Although the first health insurance law had been passed, South Korea was still struggling economically, and the government was not stable. As a result, people across the nation received little to no assistance from public health services, relying mostly on private clinics for care, especially in rural area like Buyeo.

Through the selfless acts and efforts of these pioneering physicians, including Dr. Song, South Korea saw significant improvements in public health. The average life expectancy for males increased from 51.1 years in the 1960s to 75.7 years by 2006, and for females, from 53.7 years to 82.4 years during the same period. Furthermore, infant mortality rates dropped from 61 per 1,000 live births in the 1960s to 5.3 per 1,000 in 2005 [4].

As South Korea developed and more healthcare professionals emerged, Dr. Song retired from his clinic in Buyeo in 1986, after 20 years of service. He continued his medical practice in Seoul, where he opened a small private clinic that allowed him to provide personalized care. His commitment to service extended beyond his clinic; he participated in clinical mission trips to Cambodia and Myanmar and offered free consultations to blind individuals at Sebit Church.

Dr. Song's lifelong dedication to medicine and his innovative approach to rural healthcare have left a lasting legacy.

## Conclusions

Dr. Kisu Song continued serving until June 2020, when his health declined, preventing him from continuing his work. He passed away on October 21, 2021. Whenever his grandson told him he wanted to become a doctor, the latter would always say, 'Treat patients like God's gift and treasure.' Hearing this from someone who practiced medicine for over 50 years carries profound meaning. We hope this message resonates with new generations of doctors, including ourselves, so that his legacy can be carried forward.

Dr. Song's life and career reflect a profound commitment to medical service and community welfare. From his early experiences during a time of great upheaval to his pioneering work in rural South Korea, Dr. Song's contributions have had a lasting impact on the field of medicine. His story is a powerful example of how personal resilience, combined with professional dedication, can profoundly influence the lives of others. Although Dr. Song may not have achieved global recognition through research or medical inventions, his role as a pioneer of Korean medicine, particularly in rural practice, underscores his invaluable contributions to healthcare in South Korea. His legacy continues to inspire and remind us of the essential role that dedicated individuals play in advancing the health and well-being of their communities.
